# Fenbendazole Exhibits Antitumor Activity Against Cervical Cancer Through Dual Targeting of Cancer Cells and Cancer Stem Cells: Evidence from In Vitro and In Vivo Models

**DOI:** 10.3390/molecules30112377

**Published:** 2025-05-29

**Authors:** Xi Lei, Yi Wang, Yuanyuan Chen, Jinyue Duan, Xin Gao, Zhongyi Cong

**Affiliations:** Department of Regenerative Medicine, School of Pharmaceutical Science, Jilin University, Fujin Road 1266, Changchun 130021, China; leixi2002@163.com (X.L.); chenyuanyuan1@zcsgwykhypt.wecom.work (Y.C.); duanjy18@mails.jlu.edu.cn (J.D.); gaoxin20@mails.jlu.edu.cn (X.G.)

**Keywords:** cervical cancer, cancer stem cell, fenbendazole, cell cycle

## Abstract

Cervical cancer remains a major threat to women’s health, with advanced cases often exhibiting recurrence and metastasis due to cancer stem cells driving therapy resistance. This study evaluated fenbendazole (FBZ), a repurposed veterinary anthelmintic, for its antitumor activity dual targeting cervical cancer cells (CCCs) and cervical cancer stem cells (CCSCs). CD133^+^CD44^+^ CCSCs were isolated from HeLa and C-33 A cell lines via immunomagnetic sorting and validated for stemness. Cell proliferation, cell cycle and apoptosis, and protein expression were detected by MST assay, flow cytometry, and Western blot analysis, respectively. FBZ dose-dependently inhibited proliferation, induced G2/M arrest, and triggered apoptosis in both CCCs and CCSCs. Mechanistically, FBZ upregulated cyclin B1 and phosphorylation of cdc25C-Ser198, while downregulating Wee1, phosphorylation of CDK1, and phosphorylation of cdc25C-Ser216, collectively enforcing G2/M blockade. In vivo, FBZ (100 mg/kg) significantly suppressed tumor growth in xenograft models without weight loss, contrasting with cisplatin-induced toxicity. Survival analysis revealed 100% survival in FBZ-treated mice versus 40% in cisplatin and 0% in untreated controls. These findings demonstrate FBZ’s unique ability to simultaneously target bulk tumor cells and therapy-resistant CCSCs via cell cycle disruption, supported by its preclinical safety and efficacy, positioning it as a promising therapeutic candidate for cervical cancer.

## 1. Introduction

Cervical cancer is one of the common malignant tumors in women. Its incidence rate (6.8%) and mortality rate (8.1%) both rank fourth in female malignant tumors, with 661,021 new cases and 348,189 deaths in 2022 [1]. Human papillomavirus is a major risk factor for the occurrence of this disease [2]. The three common histological types of cervical cancer are squamous cell carcinoma (70–80%), adenocarcinoma (approximately 20%), and adenosquamous carcinoma [3,4]. The treatment methods for patients with cervical cancer include surgery, radiotherapy, chemotherapy, immunotherapy, targeted therapy, or a combination of these treatments. The suitable treatment plan is based on the pathological type, tumor size, and occurrence of metastasis, as well as the patient’s age and future fertility requirements. For patients with early-stage cervical cancer, surgery is the main treatment method, with a high cure rate and relatively good prognosis. However, for advanced-stage patients, the curative options are limited [3,4]. Chemotherapy and radiotherapy, with significant toxic side effects, frequently lead to poor patient compliance and drug resistance of tumor cells for long-term administration [5]. Although immunotherapy and targeted therapy are effective in some patients with low side effects, the selection of patient indications and high costs limit the wide application of these methods [6]. A study analyzing the survival rates of 62,212 cervical cancer patients, based on the 2018 International Federation of Gynecology and Obstetrics cervical cancer staging schema, showed that the 5-year overall survival rates of patients in stage III and stage IV are approximately 40% and 20%, respectively [7]. Therefore, the development of new anti-cervical cancer drugs with high efficiency and low toxicity may provide new options for the treatment of patients with advanced cervical cancer.

The low survival rate of patients with advanced cervical cancer is closely related to tumor recurrence and chemotherapy/radiotherapy resistance [8,9]. The widely accepted perspective suggests that the development of treatment resistance and recurrence are attributed to a minor population of cancer cells, which exhibit slow proliferation rates and enhanced phenotypic and functional characteristics associated with stemness, referred to as cancer stem cells (CSCs) [10,11,12]. These CSCs are characterized by their ability to initiate tumors, sustain self-renewal, and differentiate into multiple cell types. Their sluggish cell cycle allows them to remain in a semi-dormant phase, thereby shielding them from the effects of various anti-proliferative cancer therapies. They drive the growth, invasion, migration, and immune evasion of tumor tissue [13,14,15]. At present, the isolation and identification of tumor stem cells are mainly achieved by cell surface-specific markers and biological characteristics. Drug development targeting CSCs has also received increasing attention [16,17].

Both cancer cells and CSCs exhibit significant heterogeneity [18]. The development of novel anticancer drugs demands substantial resources in terms of manpower, financial investment, and time, often accompanied by low success rates and high investment risks. Consequently, repurposing existing drugs for new therapeutic applications represents a highly promising strategy. Drug repurposing involves utilizing approved drugs to treat different diseases, which can significantly reduce treatment costs [19]. Research has revealed that tumors share certain characteristics with parasites, such as relying on glycolysis for energy metabolism, employing similar immune evasion mechanisms, and manipulating the surrounding microenvironment to promote their survival [20,21,22]. Based on these similarities, it has been hypothesized that antiparasitic drugs may possess antitumor properties. Fenbendazole (FBZ), methyl N-(6-phenylsulfanyl-1H-benzimidazol-2-yl) carbamate, a widely used broad-spectrum benzimidazole anthelmintic, is known for its low toxicity and cost-effectiveness. It is effective against a variety of gastrointestinal parasites in animals and humans [23,24,25]. FBZ exerts its effects by binding to beta-tubulin, thereby inhibiting microtubule assembly in parasites, which results in their death while minimizing harm to the host organism [26]. A randomized, placebo-controlled phase 1 first-in-human study involving 70 healthy volunteers demonstrated that oxfendazole (the primary metabolite of FBZ) was well-tolerated across escalating dose levels (0.5, 1, 3, 7.5, 15, 30, and 60 mg/kg body weight), with no reported serious adverse events or deaths. No clinically significant differences were observed between the oxfendazole and placebo groups in hematological, biochemical, or urinary parameters [27]. Recent studies have revealed that FBZ displays anticancer activity across a wide range of tumor types, such as non-small cell lung cancer, breast cancer, colorectal cancer, hepatocellular carcinoma, cervical cancer, melanoma, and leukemia, by suppressing cancer cell growth [28,29,30,31,32,33,34]. Notably, while its antitumor effects are becoming increasingly recognized, two critical gaps remain: the precise molecular mechanisms underlying FBZ’s anticancer activity are still not fully elucidated, and more importantly, its impact on CSCs—the key drivers of tumor recurrence and therapeutic resistance—remains unexplored in the current literature.

In this study, we examined the in vitro effects of FBZ on the proliferation, cell cycle, and apoptosis of human cervical cancer cells (CCCs) and cervical cancer stem cells (CCSCs), as well as its in vivo effects on cervical cancer xenografts. Additionally, we preliminarily explored the underlying mechanisms involved. This study not only provides new insights into the potential of FBZ as a repurposed anti-cervical cancer agent but also highlights its potential to target both cancer cells and CSCs, offering a promising strategy to overcome tumor heterogeneity and drug resistance.

## 2. Results

### 2.1. The Effect of FBZ on Tumor Cell Proliferation

To investigate the effects of FBZ on the proliferation of different tumor cells, human breast cancer cells MDA-MB-231 and ZR-75-1, human colon cancer cells HCT 116, and human CCCs HeLa and C-33 A were treated with FBZ at concentrations of 0.25, 0.5, 1, 2.5, 5, and 10 μM, respectively. Human fibroblast cells BJ were used as normal cell control. After 48 h of treatment, cell proliferation was detected by the MTS assay. The results showed that the proliferation of the five tumor cell lines decreased with the increase in FBZ concentration, while the proliferation of human fibroblast cells BJ remained at a high level and relatively constant (Figure S1). The IC_50_ of FBZ on HeLa cells was the lowest, which was 0.59 μM. The IC_50_ on C-33 A was 0.84 μM, MDA-MB-231 was 1.80 μM, ZR-75-1 was 1.88 μM, and HCT 116 was 3.19 μM. These results indicated that FBZ showed a strong inhibitory effect on the HeLa and C-33 A cells in vitro, so we further explored the effect of FBZ on CCSCs.

### 2.2. Isolation and Identification of CCSCs

**Figure 1:**
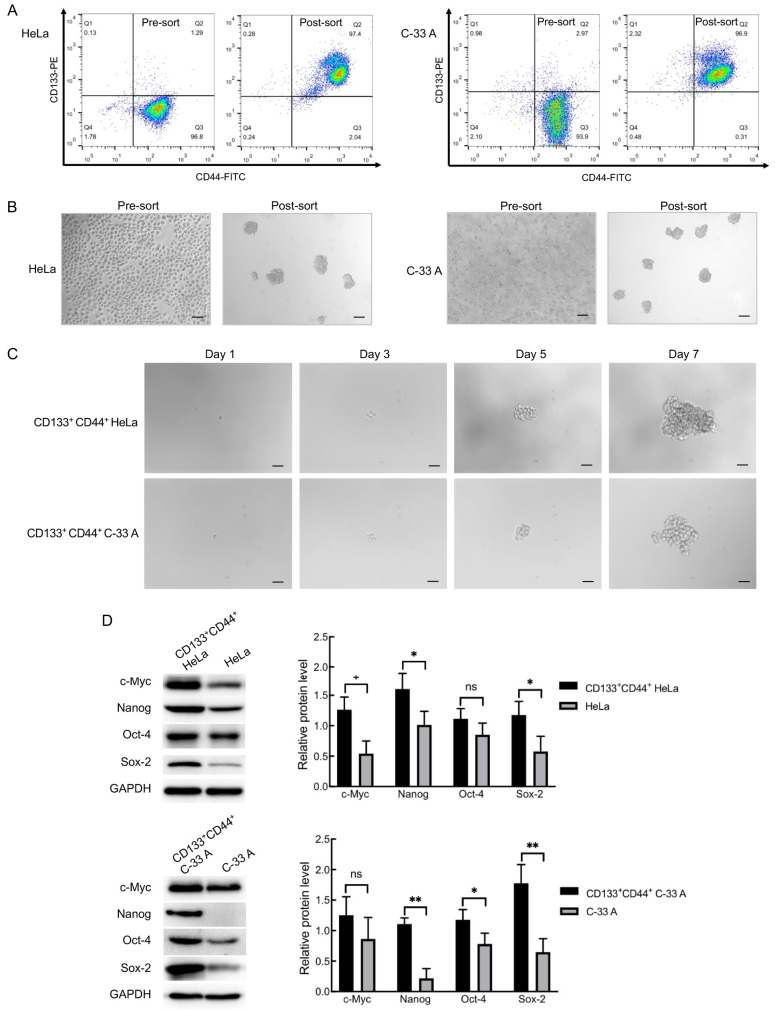
Isolation and identification of cervical cancer stem cells (CCSCs). (**A**) Detection of CD133^+^CD44^+^ cervical cancer cells by flow cytometry; (**B**) Cell morphology of CD133^+^CD44^+^ cells cultured for 5 days after being isolated from cervical cancer cells with immunomagnetic bead (bar: 100 μm); (**C**) Identification of CD133^+^CD44^+^ cervical cancer cells using single-cell colony-forming assay (bar: 50 μm); (**D**) Detection of the stemness-related markers by Western blot. * *p* < 0.05; ** *p* < 0.01; ns: no significance; *n* = 3.

As reported previously, CD133 and CD44 can be used as surface markers of CCSCs [35,36,37], therefore we isolated CD133^+^CD44^+^ cells from CCCs HeLa and C-33 A by immunomagnetic bead sorting. The initial number of CCCs was 2 × 10^7^. After sorting, 2.05 × 10^5^ (1.03%) of CD133^+^CD44^+^ HeLa cells and 1.90 × 10^5^ (0.95%) of CD133^+^CD44^+^ C-33 A cells were obtained. To detect the purity of sorted CD133^+^CD44^+^ CCCs, flow cytometry was used to detect the percentage of CD133 and CD44 positive cells before and after sorting. As shown in Figure 1A, there were 1.29% of CD133^+^CD44^+^ cells in pre-sort HeLa cells, while the post-sort purity was 97.4%. As regards the C-33 A cells, there were 2.97% of CD133^+^CD44^+^ cells before sorting, and 96.9% after sorting. The sorted cells were cultured in serum-free DMEM/F12 medium (supplemented with EGF, bFGF, and B27). Observed under a microscope, the CD133^+^CD44^+^ HeLa cells and C-33 A cells grew in suspension and appeared as irregular cell clusters (Figure 1B).

To confirm the stem-like properties of the isolated CD133^+^CD44^+^ cell population, we assessed two key CSC features: single-cell colony-forming capacity, and expression of stemness-related markers (Oct-4, Sox-2, Nanog, and c-Myc). As shown in Figure 1C, the single CD133^+^CD44^+^ cell proliferated, gradually forming an irregular spherical colony, and the cell cluster gradually increased with time. The expression of stem cell-specific genes Oct-4, Sox-2, Nanog, and c-Myc of CD133^+^CD44^+^ HeLa and C-33 A cells was detected by Western blot. Compared with pre-sort HeLa cells, the expression of c-Myc, Nanog, and Sox-2 was significantly increased in the CD133^+^CD44^+^ HeLa cells (*p* < 0.05, Figure 1D. The uncropped Western blot raw images are provided in Figure S2). Similarly, the expression of Nanog, Oct-4, and Sox-2 in the CD133^+^CD44^+^ C-33 A cells was higher than that of the pre-sort cells (*p* < 0.05).

In summary, the CD133^+^CD44^+^ cells isolated from the HeLa and C-33 A cells are CCSCs with single-cell colony-forming capacity and high expression of stemness-related markers. These two cell populations are termed HeLa SCs and C-33 A SCs (HeLa stem cells and C-33 A stem cells, respectively) throughout this study.

### 2.3. The Effect of FBZ on Proliferation, Cell Cycle, and Apoptosis of CCCs and CCSCs

**Figure 2:**
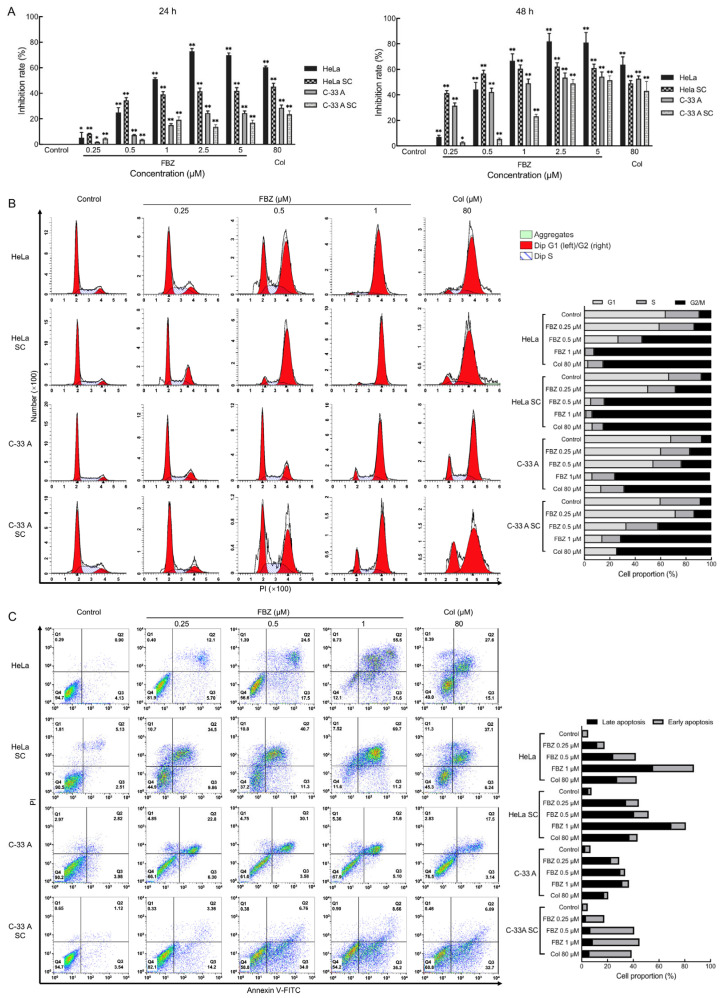
The effect of fenbendazole (FBZ) on proliferation, cell cycle, and apoptosis of cervical cancer cells (CCCs) and CCSCs. (**A**) The effect of different concentrations of FBZ treatment on the proliferation of CCCs and CCSCs (MTS assay). * *p* < 0.05 vs Control; ** *p* < 0.01 vs. Control; *n* = 3. (**B**) PI staining for detecting changes in cell cycle of CCCs and CCSCs after 24-h FBZ treatment (flow cytometry). The bar chart represents the proportion of cells in each stage of the cell cycle. (**C**) Annexin V-FITC/PI staining for detecting apoptosis in CCCs and CCSCs after 48-h FBZ treatment (flow cytometry). The bar chart represents the proportion of apoptotic cells in each group. SC: stem cell; Col: colchicine.

The HeLa, HeLa SC, C-33 A, and C-33 A SC cells were treated with different concentrations of FBZ for 24 and 48 h, and cell proliferation was detected by the MTS method. Colchicine (Col) was used as a positive control. The results demonstrated significant antiproliferative effects of FBZ on both CCCs and CCSCs across a concentration range of 0.25–5 μM (all *p* < 0.05 vs. control, with maximal inhibition achieving *p* < 0.01 at higher concentrations; Figure 2A). Notably, prolonged 48-h exposure to FBZ resulted in enhanced growth suppression compared to 24-h treatment at equivalent concentrations.

To investigate the effects of FBZ on cell cycle progression in CCCs and CCSCs, HeLa, HeLa SC, C-33 A, and C-33 A SC cells were treated with FBZ (0.25, 0.5, and 1 μM) for 24 h, followed by PI staining and flow cytometric analysis. The results revealed that FBZ induced dose-dependent cell cycle arrest in both CCCs and CCSCs, as evidenced by progressively increased G2/M phase populations with escalating FBZ concentrations. At the highest concentration tested (1 μM), FBZ treatment resulted in remarkable G2/M accumulation, with >90% of HeLa and HeLa SC cells and >70% of C-33 A and C-33 A SC cells arrested in the G2/M phase (Figure 2B).

To evaluate the effect of FBZ on the apoptosis of CCCs and CCSCs, HeLa, HeLa SC, C-33 A, and C-33 A SC cells were treated with 0.25, 0.5, and 1 μM FBZ for 48 h. Cell apoptosis was assessed by Annexin V-FITC/PI dual staining followed by flow cytometry. As demonstrated in Figure 2C, FBZ treatment induced concentration-dependent apoptosis in both CCCs and CCSCs. At the maximal tested concentration (1 μM), FBZ elicited profound apoptotic effects, resulting in over 80% apoptosis in the HeLa and HeLa SC cells, while the C-33A and C-33A SC cells exhibited approximately 40% apoptosis. These results demonstrate that FBZ exerts inhibitory effects on the proliferation of CCCs and CCSCs, induces cell cycle arrest, and promotes apoptosis in vitro.

### 2.4. Mechanistic Investigation of FBZ Antitumor Activity

**Figure 3:**
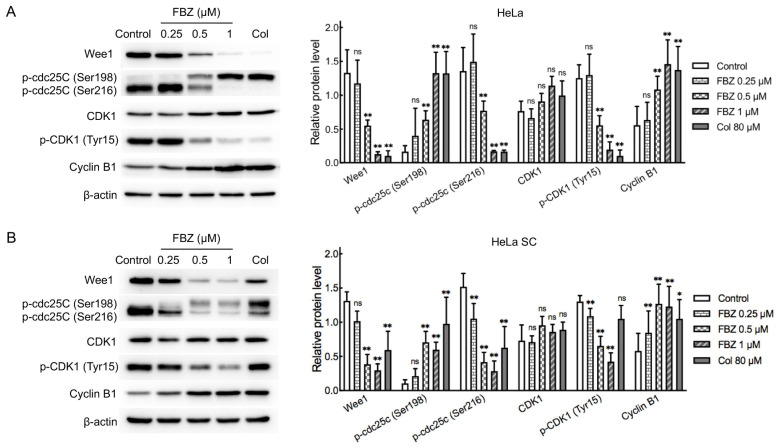
Detection of cell cycle-associated proteins by Western blot. (**A**) HeLa cell; (**B**) HeLa SC. * *p* < 0.05 vs. Control; ** *p* < 0.01 vs. Control; ns: no significance vs. Control; *n* = 3. CDK1: cyclin-dependent kinase 1.

Benzimidazole anthelmintics primarily exert their therapeutic effects through inhibition of tubulin polymerization, a process critically involved in mitotic spindle formation during M phase progression [26]. Based on the aforementioned results of cell proliferation and cell cycle analysis in CCCs and CCSCs, FBZ exhibited stronger inhibitory effects on HeLa and HeLa SCs compared to C-33 A and C-33 A SCs (Figure 2A,B). Therefore, we selected HeLa cells and HeLa SCs for further investigation into the underlying mechanisms of FBZ action. We analyzed alterations in key regulatory factors (Wee1, cdc25C, CDK1, and cyclin B1), controlling the G2/M checkpoint. The cell cycle kinase CDK1 plays a pivotal role in G2/M phase transition through its association with cyclin B1 to form the CDK1-cyclin B1 complex. CDK1 activity is precisely controlled by opposing regulatory mechanisms: inhibitory phosphorylated at Tyr15 by Wee1 versus activating dephosphorylated by cdc25C [38]. Importantly, cdc25C itself is subject to regulatory phosphorylation, with stimulatory effects mediated by Ser198 phosphorylation and inhibitory effects through Ser216 phosphorylation [39,40]. As demonstrated in Figure 3 (the uncropped Western blot raw images are provided in Figure S2), FBZ treatment (0.5 μM and 1 μM) for 24 h elicited concordant alterations in the G2/M checkpoint regulators across both HeLa cells and HeLa SCs. Quantitative analysis revealed a marked upregulation of cyclin B1 protein expression (*p* < 0.01), a significant reduction in CDK1 phosphorylation at Tyr15 (*p* < 0.01), downregulation of Wee1 levels (*p* < 0.01), enhanced phosphorylation of cdc25C at Ser198 (*p* < 0.01), and diminished phosphorylation at Ser216 (*p* < 0.01). The data demonstrated that FBZ treatment promoted activation of the CDK1-cyclin B1 complex and induced significant G2 phase accumulation in both CCCs and CCSCs, findings that were concordant with our prior cell cycle analysis results.

### 2.5. The Effect of FBZ on Cervical Cancer Xenografts

**Figure 4:**
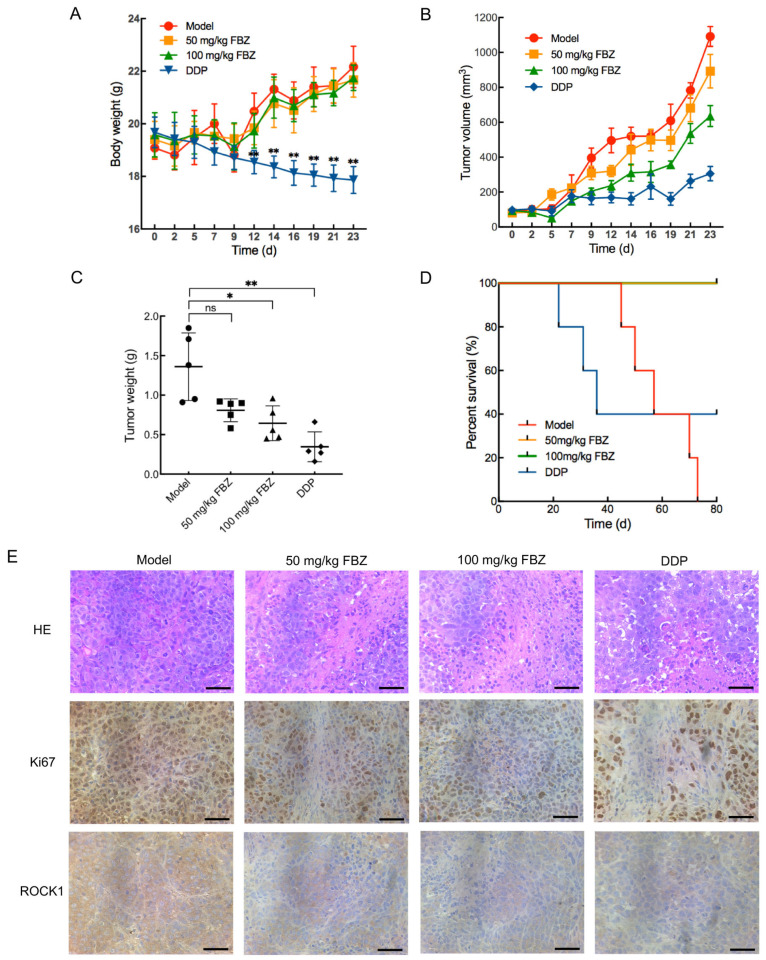
The effect of FBZ on cervical cancer xenografts. The xenograft models were established using female BALB/c/nu nude mice implanted with HeLa CCCs. The tumor-bearing nude mice were randomly divided into four groups: Model, 50 mg/kg FBZ, 100 mg/kg FBZ, and cisplatin (DDP). The Model and FBZ groups were administered once a day for a total of 23 days, and the DDP group was administered twice a week for a total of 23 days. (**A**) Body weight (*n* = 5); (**B**) Tumor volume (*n* = 5); (**C**) Tumor weight at the end of treatment (*n* = 5); (**D**) Survival analysis over an 80-day observation period following tumor cell inoculation (*n* = 5); (**E**) Hematoxylin-Eosin (HE) and immunohistochemical staining of tumor tissue slices (bar: 50 μm). * *p* < 0.05 vs. Model; ** *p* < 0.01 vs. Model; ns: no significance vs. Model; *n* = 5. ROCK1: Rho-associated coiled-coil containing protein kinase.

To evaluate the in vivo antitumor efficacy of FBZ against cervical cancer, we established xenograft models using female BALB/c/nu nude mice implanted with HeLa CCCs. Tumor-bearing mice were randomized into four groups: Model, 50 mg/kg FBZ, 100 mg/kg FBZ, and DDP (cisplatin as positive control). Throughout the entire experimental period, FBZ-treated mice exhibited normal alertness and spontaneous locomotor activity, displayed smooth fur, maintained steady respiration, and showed no ocular/nasal/oral secretions, with normal food intake and defecation patterns. Longitudinal monitoring revealed no significant body weight changes in the FBZ-treated groups compared to the Model group, whereas DDP treatment induced significant weight reduction at multiple time points (days 12, 14, 16, 19, 21, and 23; *p* < 0.01, Figure 4A). At the end of the treatment period, 5 mice from each group were euthanized. Gross anatomical examination revealed no macroscopic signs of congestion, swelling, or atrophy in major organs (heart, liver, spleen, lungs, and kidneys). The organ coefficients (organ-to-body weight ratios) were calculated for all experimental groups. Compared with the Model group, no statistically significant differences were observed in the heart, liver, lung, or kidney coefficients of either the 50 mg/kg FBZ group, 100 mg/kg FBZ group, or DDP group (Figure S3). While the splenic coefficient showed no significant change in the 50 mg/kg FBZ group, both the 100 mg/kg FBZ group (*p* < 0.05) and DDP group (*p* < 0.01) exhibited significant reductions in the spleen coefficient, which might potentially correlate with tumor-bearing immune responses, and the precise mechanism requires further investigation. Collectively, these findings demonstrate that FBZ treatment did not induce severe toxic side effects in tumor-bearing mice.

Tumor volume analysis revealed differential treatment effects, with the 50 mg/kg FBZ group showing no significant reduction compared to the model controls, whereas 100 mg/kg FBZ treatment induced statistically significant tumor regression at multiple time points (days 5, 9, 12, 19, and 21: *p* < 0.05; day 23: *p* < 0.01; Figure 4B), paralleled by DDP-induced tumor suppression (days 12 and 16: *p* < 0.05; days 9, 14, 19, 21, and 23: *p* < 0.01).

Tumor weight measurements at the end of treatment demonstrated that the 100 mg/kg FBZ (*p* < 0.05) and DDP (*p* < 0.01) groups exhibited significant reductions versus the Model group, while the 50 mg/kg FBZ group showed no statistically significant difference in tumor weight compared to the Model group (*p* > 0.05, Figure 4C). Quantitative analysis yielded tumor inhibition rates of (40.6 ± 10.6)%, (52.6 ± 16.2)%, and (74.6 ± 13.9)% for the 50 mg/kg FBZ, 100 mg/kg FBZ, and DDP groups respectively, collectively establishing FBZ’s significant growth-inhibitory effects on cervical cancer xenografts.

Survival analysis over an 80-day observation period following tumor cell inoculation revealed striking differences among treatment groups: while all mice in the Model group succumbed, the DDP group showed 40% survival. In contrast, both FBZ-treated groups (50 and 100 mg/kg) exhibited 100% survival throughout the study duration, demonstrating statistically significant lifespan extension compared to the Model (*p* < 0.01) and DDP (*p* < 0.05) groups (Figure 4D). These findings conclusively demonstrate FBZ’s ability to prolong survival in tumor-bearing nude mice.

In addition, histopathological analysis via Hematoxylin-Eosin (HE) staining revealed distinct morphological differences among treatment groups: tumor tissues from the Model group exhibited tightly packed cellular architecture with hyperchromatic, enlarged nuclei, whereas both FBZ-treated groups (50 and 100 mg/kg) and the DDP group displayed characteristic features of therapeutic response, including disorganized cellular arrangement, loss of nuclear uniformity, and evident necrotic foci (Figure 4E). Complementary immunohistochemical evaluation of proliferation marker Ki67 and metastatic regulator Rho-associated coiled-coil containing protein kinase (ROCK1) demonstrated significant downregulation of both biomarkers in all treatment groups compared to untreated controls. Collectively, these histopathological and molecular findings substantiate that FBZ induces tumor necrosis while exerting dual antiproliferative and antimetastatic effects in cervical cancer xenografts.

## 3. Discussion

Cervical cancer is a common gynecological malignancy, and the current treatment strategies primarily involve a comprehensive approach combining surgery and radiotherapy, with chemotherapy as an adjunct [3]. For patients with advanced-stage disease who are ineligible for surgery, palliative care is the main option, often resulting in poor prognosis [4]. Tumor recurrence and metastasis are the leading causes of mortality in clinical settings [8,9]. It is widely accepted that CSCs play a critical role in driving tumor growth, invasion, migration, and drug resistance [10,12]. This study provides compelling preclinical evidence that FBZ, a well-established veterinary anthelmintic with decades of proven clinical safety [24], exerts potent antitumor activity against both human CCCs and CCSCs in vitro, while significantly inhibiting xenograft tumor progression in vivo with minimal observed toxicity, suggesting its strong potential as a repurposed therapeutic candidate for cervical cancer that could offer substantial economic advantages over de novo drug development.

Building upon existing pharmacological foundations, we evaluated the inhibitory effects of FBZ on tumor cell proliferation in vitro. Our results demonstrated that FBZ significantly suppressed the proliferation of multiple human cancer cell lines, including breast cancer (MDA-MB-231 and ZR-75-1), colon cancer (HCT 116), and cervical cancer (HeLa and C-33 A) cells, with particularly potent growth inhibition observed in HeLa cells, whereas no significant cytotoxic effect was detected in normal human fibroblasts (BJ cell line) even at the highest tested concentration. These findings suggest that FBZ exhibits broad-spectrum and selective antitumor efficacy. Current research on the antitumor effects of FBZ has primarily focused on its ability to inhibit tumor cell proliferation [28,29,30,31,32,33,34], while its impact on CSCs remains unexplored. CD133, CD44, CD24, CD71, and Brcp1 were commonly used markers for isolating CCSCs [35,36,37,41,42]. In the present study, CCSCs were successfully isolated using CD133 and CD44 as surface markers. These isolated CCSCs demonstrated characteristic stem cell properties, including the ability to form single-cell-derived clones in serum-free culture conditions and elevated expression of key stemness markers (Oct-4, Sox-2, Nanog, and c-Myc). CSCs, defined by their expression of stemness-related genes, are widely recognized as pivotal mediators of tumor initiation, disease progression, therapeutic resistance, and metastatic dissemination in malignant neoplasms. These properties are largely attributed to their capacity for self-renewal, multilineage differentiation, intrinsic drug resistance, and metastatic potential [14,15,43]. Notably, our findings demonstrate that FBZ exerts dual inhibitory effects on both CCSs and CCSCs. Specifically, FBZ treatment resulted in the suppression of cellular proliferation, G2/M cell cycle arrest, and the induction of apoptosis. These results suggest that FBZ’s ability to simultaneously target both differentiated tumor cells and the CSC population may provide significant therapeutic advantages by potentially overcoming three major clinical challenges: tumor recurrence, metastatic spread, and acquired drug resistance. However, additional mechanistic studies are warranted to fully elucidate these effects.

FBZ exerts its parasiticidal effects through the inhibition of microtubule polymerization in parasites, thereby disrupting the mitotic processes of cells [26]. Through cell cycle analysis, we observed a pronounced G2/M phase accumulation in both CCCs and CCSCs upon FBZ exposure. Wee1, cdc25C, CDK1, and cyclin B1 are key regulatory proteins of the mammalian cell cycle, primarily functioning during the G2/M phase. CDK1 (also known as cdc2) is a central kinase in cell cycle regulation, forming a complex with cyclin B1 (CDK1-cyclin B1) to drive the transition from the G2 to M phase. Cyclin B1, the regulatory subunit of CDK1, gradually accumulates during the G2 phase, and binds to and activates CDK1. Upon entering the M phase, cyclin B1 is ubiquitinated and degraded, leading to CDK1 inactivation and the completion of mitosis. The activity of CDK1 is regulated by both Wee1 and cdc25C. Wee1 inhibits CDK1 activity by phosphorylating Tyr15, whereas cdc25C activates CDK1 through dephosphorylation [38]. Our study revealed consistent changes in these cell cycle-related proteins in both CCCs and CCSCs following FBZ treatment. Specifically, FBZ treatment significantly increased cyclin B1 expression, reduced CDK1 phosphorylation levels, decreased Wee1 expression, elevated phosphorylation at Ser198 of cdc25C, and reduced phosphorylation at Ser216 of cdc25C. Cyclin B1 transcription begins in the S phase, with expression peaking during the G2 phase [44]. The marked increase in cyclin B1 levels indicates a large population of cells arrested in the G2 phase. Although CDK1 expression remained unchanged, its phosphorylation levels were significantly reduced, suggesting that the CDK1-cyclin B1 complex was in an activated state, further confirming the G2/M phase arrest. Phosphorylation at Ser198, a positive regulatory site of cdc25C, promotes its activation, whereas phosphorylation at Ser216, a negative regulatory site, inhibits its activity [39,40]. In the present study, FBZ treatment significantly increased the phosphorylation level of Ser198 and decreased the phosphorylation level of Ser216 in cdc25C, thereby promoting its activation. Additionally, the expression of Wee1 was markedly reduced, leading to enhanced dephosphorylation and activation of CDK1. Consequently, FBZ treatment led to an accumulation of CCCs and CCSCs in the G2/M phase. Previous study has demonstrated that during G2/M phase arrest, activation of the CDK1-cyclin B1 complex induces phosphorylation of Bcl-2 family proteins, attenuating their anti-apoptotic function. This subsequently activates Bak, triggering the mitochondrial apoptotic pathway and ultimately leading to cell apoptosis [45]. Our findings reveal that FBZ not only induces G2/M phase arrest in both CCCs and CCSCs, but also promotes apoptosis in these cell populations, potentially through this mechanistic pathway. Notably, since G2/M phase-arrested cells exhibit heightened sensitivity to both radiotherapy and chemotherapy [46,47], FBZ, with its potent cell cycle arrest capabilities, holds promise as a radiosensitizer or chemosensitizer in clinical settings, pending thorough investigation of its safety and efficacy.

To further evaluate the therapeutic potential of FBZ against cervical cancer in vivo, we established the xenograft mouse model. Pharmacological assessment revealed that FBZ administration at 100 mg/kg elicited significant tumor growth inhibition. While DDP-treated animals exhibited maximal tumor suppression, this group manifested substantial body weight loss, indicative of marked treatment-related toxicity. Conversely, FBZ-treated groups (50 and 100 mg/kg) maintained stable body weights comparable to the Model group, demonstrating superior tolerability. Throughout the entire treatment period, FBZ-treated mice maintained normal alertness and spontaneous activity, showed steady respiration, exhibited smooth fur without discharge from the eyes/nose/mouth, and demonstrated normal feeding and defecation patterns. Gross pathological examination after euthanasia revealed no macroscopic signs of congestion, swelling, or atrophy in major organs. Organ coefficient (heart, liver, spleen, lungs, and kidneys) measurements showed no significant differences compared with controls, except for significantly reduced spleen coefficients in the 100 mg/kg FBZ group and DDP group—a phenomenon potentially related to tumor-bearing immune responses. Collectively, these findings demonstrate that FBZ treatment did not induce severe toxic side effects. In addition, published preclinical and clinical studies have consistently demonstrated good tolerability of FBZ treatment without significant toxicity/side effects [48,49]. Kaplan–Meier survival analysis showed 100% survival in both FBZ treatment groups throughout the study duration, contrasting with 40% survival in the DDP cohort and universal mortality in untreated tumor-bearing mice. These findings establish that FBZ not only extends survival in tumor-bearing hosts but does so with an exceptional safety margin, as corroborated by body weight trajectories. Histopathological evaluation through HE staining and immunohistochemical profiling demonstrated that FBZ treatment induced extensive tumor necrosis while suppressing proliferative (Ki67) and metastatic (ROCK1) markers in xenograft tissues. Collectively, these preclinical data substantiate FBZ as a promising therapeutic candidate for cervical cancer, combining robust antitumor efficacy with an advantageous safety profile.

## 4. Materials and Methods

### 4.1. Animals

Female BALB/c nude mice, aged 4–6 weeks and weighing (16 ± 2) g, were procured from Beijing Huafukang Biotechnology Co., Ltd. (Beijing, China) and housed within the Specific Pathogen Free level barrier system of the Laboratory Animal Center, School of Public Health, Jilin University, Changchun, China. Animal feeding conditions, environment, and experimental procedures were carried out in accordance with the protocols approved by the Institutional Animal Care and Use Committee of School of Pharmaceutical Science, Jilin University (No. 20210031).

### 4.2. Cell Lines

The human cervical cancer cell line C-33 A and human breast cancer cell line ZR-75-1 were purchased from National Collection of Authenticated Cell Cultures (Shanghai, China). The human cervical cancer cell line HeLa, human breast cancer cell line MDA-MB-231, human colon cancer cell line HCT 116, and human fibroblast cell line BJ were preserved in our laboratory. These cells were cultured in RPMI 1640 medium (Gibco, Thermo Fisher Scientific Inc., Shanghai, China) supplemented with 10% fetal bovine serum (FBS, Gibco) at 37 °C in a humidified atmosphere containing 5% CO_2_. Cells in logarithmic growth phase were collected for the experiments.

### 4.3. Cell Proliferation Assay

Cell proliferation was assessed using the CellTiter 96^®^ AQueous One Solution Cell Proliferation Assay kit (MTS, Promega Corporation, Beijing, China). The cells were seeded in a 96-well plate at 5 × 10^3^ cells/well in 100 μL and incubated at 37 °C with 5% CO_2_ for 24 h. Then 100 μL of FBZ (Sigma-Aldrich, Shanghai, China) solution was added to each well to achieve final concentrations of 0.25, 0.5, 1, 2.5, 5, and 10 μM. Each concentration was triplicated. Dimethyl sulfoxide (DMSO, Sigma-Aldrich) was added as solvent control, and the amount of DMSO added to each group was the same, except for the blank group with 100 μL of culture medium added. The cells were cultivated for another 48 h. Then 20 μL of MTS was added to each well, and incubated with the cells at 37 °C in the dark for 1–4 h. The absorbance (A) value at 490 nm of each well was detected by a microplate reader (Perlong, Beijing, China). The inhibition rate was calculated using the following formula.

Inhibition rate = (A_control_ − A_sample_)/(A_control_ − A_blank_) × 100%. (1)

The half-maximal inhibitory concentration (IC_50_) of FBZ was calculated using the software GraphPad Prism, version 8.4.3.

### 4.4. Isolation of CD133^+^CD44^+^ Cells from CCCs

The CD133^+^CD44^+^ cells were isolated from CCCs using the CELLection^TM^ Biotin Binder Kit (Invitrogen, Thermo Fisher Scientific Inc.) According to the manufacturer’s instructions, 2 × 10^7^ of cervical cancer cells were incubated sequentially with the prepared CD44 antibodies (eBioscience, Thermo Fisher Scientific Inc.)-coated Dynabeads, and CD133 antibodies (eBioscience)-coated Dynabeads at 4 °C for 20 min. The CD133^+^CD44^+^ cells were then sorted by a magnet. After being released from the beads, the obtained CD133^+^CD44^+^ cells were cultivated in DMEM/F12 medium (Gibco) supplemented with 2% B27 (Gibco), 20 ng/mL of EGF, and 20 ng/mL of bFGF (both from PeproTech, Thermo Fisher Scientific Inc.) at 37 °C with 5% CO_2_.

### 4.5. Flow Cytometry Detection of Cell Surface Molecules

The cultivated CCCs and CD133^+^CD44^+^ cells were collected respectively, digested into single cells, and washed with ice-cold PBS. Then, 1 × 10^6^ cells were resuspended in 100 μL of ice-cold PBS containing 2% FBS, and 2 μL of FITC-CD44 (eBioscience) antibody and 5 μL of PE-CD133 antibody (eBioscience) were added into the cell suspensions, followed by incubation at 4 °C in the dark for 40 min. After being washed twice with 1 mL of ice-cold PBS containing 2% FBS, the cells were resuspended in 400 μL of ice-cold PBS containing 2% FBS, and analyzed using the BD FACSCalibur Flow Cytometer (BD Biosciences, San Jose, CA, USA) within 1 h.

### 4.6. Colony Formation Assay

The cultivated CD133^+^CD44^+^ cells were collected and digested into single cells. After the cell density was adjusted to 5 cells/mL, the cell suspension was added to a 96-well plate (100 μL/well). The cells were incubated at 37 °C with 5% CO_2_ for 7 days, and observed under a microscope (Olympus Corporation, Tokyo, Japan) every day.

### 4.7. Western Blot Analysis

The cells from different treatment groups were harvested and lysed with RIPA lysis buffer (Dingguo Changsheng, Beijing, China). The protein concentration of each sample was determined using the BCA Protein Assay Kit (Beyotime, Shanghai, China). The proteins were separated by sodium dodecyl sulfate polyacrylamide gel electrophoresis and transferred to a polyvinylidene fluoride membrane. After being blocked with 5% skim milk, the membrane was incubated with primary antibodies overnight at 4 °C. The primary antibodies used in this study include c-Myc (1:1000, Abcam, Shanghai, China), Nanog (1:1000, Abcam), Oct-4 (1:1000, Abcam), Sox-2 (1:1000, Abcam), GAPDH (1:2000, Beyotime), Wee1 (1:1000, CST, Shanghai, China), p-cdc25C (1:1000, CST), cdc2 (CDK1, 1:1000, CST), p-cdc2 (p-CDK1, 1:1000, CST), cyclin B1 (1:1000, CST), and β-actin (1:2000, Beyotime). The membrane was then incubated with horseradish peroxidase-conjugated goat anti-rabbit IgG antibodies (1:10,000, Beyotime) for 1 h at room temperature. The target protein signals were detected using a super ECL detection reagent (Yeasen, Shanghai, China), and viewed by the Tanon Imaging System (Tanon, Shanghai, China).

### 4.8. Cell Cycle and Apoptosis Analysis

Cell cycle and apoptosis were analyzed using the Cell Cycle Analysis Kit and the Annexin V-FITC/PI Apoptosis Detection Kit (both from KeyGEN BioTECH, Nanjing, China), respectively, according to the manufacturer’s introductions. Briefly, the cells were digested into single cells and seeded in 6-well plates (3 × 10^5^ cervical cancer cells and 5 × 10^5^ CCSCs in 4 mL, respectively). After cultivation at 37 °C with 5% CO_2_ for 24 h, FBZ was added into different wells at final concentrations of 0.25, 0.5, and 1 μM. Col (80 μM [50], MedChemExpress, Shanghai, China) and DMSO were added as positive control and solvent control, respectively. The amounts of DMSO were equal in the control and FBZ-treated groups. To detect cell cycle, the cells were cultivated for another 24 h. The cells were then harvested, washed with ice-cold PBS, and fixed with ice-cold 75% ethanol at 4 °C overnight. After being washed with PBS, the cells were resuspended with 500 μL/1 × 10^6^ cells of PI staining buffer containing RNase for 30 min at room temperature in darkness for flow cytometry analysis. To detect cell apoptosis, the cells were treated with FBZ for 48 h, washed with PBS, and resuspended with 200 μL/1 × 10^5^ cells of binding buffer. After 5 μL of Annexin V-FITC was added and incubated at room temperature in the dark for 15 min, 3 μL of PI was added and incubated for another 5 min. Then 400 μL of binding buffer was added for flow cytometry detection. For analyzing cell cycle and apoptosis, standard flow cytometry procedures were used (BD FACSCalibur Flow Cytometer, BD Biosciences, San Jose, CA, USA).

### 4.9. In Vivo Xenograft Tumor Experiments

After one week of adaptive feeding, each BALB/c nude mouse was subcutaneously inoculated with 5 × 10^6^ HeLa cells in 100 μL of PBS containing 20% of Matrigel matrix (Corning Incorporated, Corning, NY, USA) into the right thigh. When tumor volumes reached approximately 50–150 mm^3^, 40 tumor-bearing nude mice were randomly divided into 4 groups (*n* = 10 per group), with 5 mice allocated for post-treatment biomarker analysis and tumor tissue collection, and the remaining 5 mice reserved for survival observation. The groups included Model (untreated control), 50 mg/kg FBZ, 100 mg/kg FBZ, and DDP groups. The mice in the Model group were administered 0.2 mL of 0.5% sodium carboxymethyl cellulose (CMC, Sinopharm Chemical Reagent Co., Ltd., Shanghai, China) orally once a day. The mice in the 50 mg/kg FBZ and 100 mg/kg FBZ groups were administered 50 and 100 mg/kg FBZ, respectively, in 0.2 mL of 0.5% CMC orally once a day. The mice in the DDP group were administered 3 mg/kg of DDP (Qilu Pharmaceutical, Jinan, China) in 0.2 mL of saline by intraperitoneal injection twice a week. All treatments were administered for 23 consecutive days. The body weight and tumor volume of mice were recorded during the process. After treatment, 5 mice from each group were euthanized to obtain the tumor tissues, which were weighed and fixed with 4% paraformaldehyde at room temperature for subsequent histology examination. Meanwhile, the major organs (hearts, livers, spleens, lungs, and kidneys) were harvested, rinsed with physiological saline, blotted dry with filter paper, and weighed. The survival times of the other 5 mice in each group within 80 days from the inoculation of tumor cells were observed. The calculation formulas for tumor volume, tumor inhibition rate, and organ coefficient are as follows.

Tumor volume (mm^3^) = 0.5 × longest diameter × (shortest diameter)^2^ (2)

Tumor inhibition rate = (1 − tumor weight of treated mouse/mean tumor weight of Model group) × 100%(3)

Organ coefficient = (organ weight/body weight) × 100%(4)

### 4.10. HE and Immunohistochemistry Staining

The tumor tissues were fixed with 4% paraformaldehyde, embedded in paraffin, and processed into 4 μm tissue sections. The sections were dewaxed with xylene and rehydrated with ethanol solutions at gradient concentrations. For HE staining, the sections were consequently stained with hematoxylin and eosin. For immunohistochemistry staining, the rehydrated sections were treated with boiling Tris-EDTA (pH 9.0) for 15 min for antigen repair, incubated with 3% H_2_O_2_ solution for 10 min to inactivate endogenous peroxidase, and blocked with non-immune serum for 10 min. Then the sections were incubated with Ki67 antibodies (1:200) and ROCK1 antibodies (1:100, both from Zenbio, Chengdu, China) at 4 °C overnight, followed by incubation with the biotin-goat anti-rabbit antibodies (1:500) and HRP-streptavidin (both from Beyotime) solution. The DAB Horseradish Peroxidase Color Development Kit (Beyotime) was used for coloration.

### 4.11. Statistical Analysis

The data are expressed as mean ± standard error of measurement. The statistical analysis and graphics were completed using the software GraphPad Prism 8.4.3. Student’s *t*-test was used to analyze the differences between the two groups, and *p* < 0.05 was considered to be statistically significant.

## 5. Conclusions

Our study demonstrates that FBZ exhibits multifaceted antitumor activity against both CCCs and CCSCs. The compound dose-dependently inhibits cellular proliferation, induces G2/M arrest, and promotes apoptosis in vitro. These in vitro observations were corroborated by in vivo studies showing potent suppression of xenograft tumor growth and the significant extension of survival in tumor-bearing mice, with no observable treatment-related toxicity. Mechanistically, FBZ appears to exert its anticancer effects through cell cycle modulation. It can upregulate cyclin B1 and phosphorylation of cdc25C-Ser198, while downregulating Wee1, phosphorylation of CDK1, and phosphorylation of cdc25C-Ser216, collectively enforcing G2/M blockade in both CCCs and CCSCs. Taken together, these preclinical findings position FBZ as a promising candidate for further development as either a primary therapeutic or adjuvant treatment modality for cervical cancer, particularly given its dual activity against both differentiated tumor cells and therapy-resistant CSCs.

## Data Availability

All the experimental data from this study are available from the corresponding author upon reasonable request.

## Supplementary Materials

The following supporting information can be downloaded at: https://www.mdpi.com/article/10.3390/molecules30112377/s1. Figure S1: The effects of FBZ on the viability of tumor cells and BJ cells; Figure S2: The original uncropped Western blot images corresponding to the results presented in Figure 1 and Figure 3; Figure S3: The organ coefficients of mice in each group after treatment.

## Abbreviations

The following abbreviations are used in this manuscript:

A	absorbance
bFGF	basic fibroblast growth factor
CCC	cervical cancer cell
CCSC	cervical cancer stem cell
CDK1	cyclin-dependent kinase 1
CMC	sodium carboxymethyl cellulose
Col	colchicine
CSC	cancer stem cell
DDP	cisplatin
DMSO	dimethyl sulfoxide
EGF	epidermal growth factor
FBS	fetal bovine serum
FBZ	fenbendazole
FITC	fluorescein isothiocyanate
HE	Hematoxylin-Eosin
IC_50_	half-maximal inhibitory concentration
MTS	3-(4,5-dimethylthiazol-2-yl)-5-(3-carboxymethoxyphenyl)-2-(4-sulfophenyl)-2H-tetrazolium
PI	propidium iodide
ROCK1	Rho-associated coiled-coil containing protein kinase
SC	stem cell
