# Supplementary material for: Fenbendazole Exhibits Antitumor Activity Against Cervical Cancer Through Dual Targeting of Cancer Cells and Cancer Stem Cells: Evidence from In Vitro and In Vivo Models
Source: Molecules. 2025 May 29;30(11):2377. doi: 10.3390/molecules30112377 (PMC12156427; doi:10.3390/molecules30112377)
Supplement: Supplementary file 1 [file molecules-30-02377-s001.zip › Figure S1.pdf]

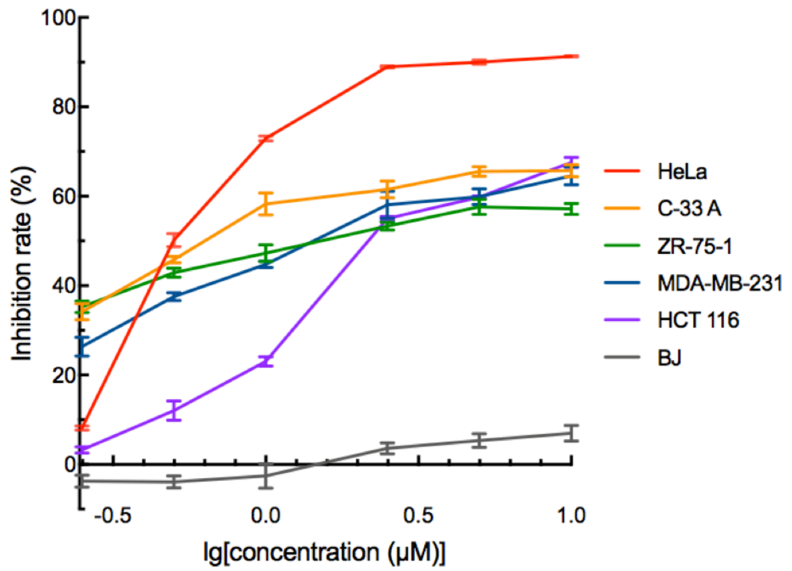

Figure S1. The effects of FBZ on the viability of tumor cells and BJ cells. Human breast cancer cells MDA-MB-231 and ZR-75-1, human colon cancer cells HCT 116, and human cervical cancer cells HeLa and C-33 A were treated with FBZ at concentrations of 0.25, 0.5, 1, 2.5, 5 and 10 μM for 48 h respectively. Human fibroblast cells BJ were used as normal cell control. Cell viability was detected by the MTS assay.
