# Supplementary material for: Fenbendazole Exhibits Antitumor Activity Against Cervical Cancer Through Dual Targeting of Cancer Cells and Cancer Stem Cells: Evidence from In Vitro and In Vivo Models
Source: Molecules. 2025 May 29;30(11):2377. doi: 10.3390/molecules30112377 (PMC12156427; doi:10.3390/molecules30112377)
Supplement: Supplementary file 1 [file molecules-30-02377-s001.zip › Figure S2.pdf]

A

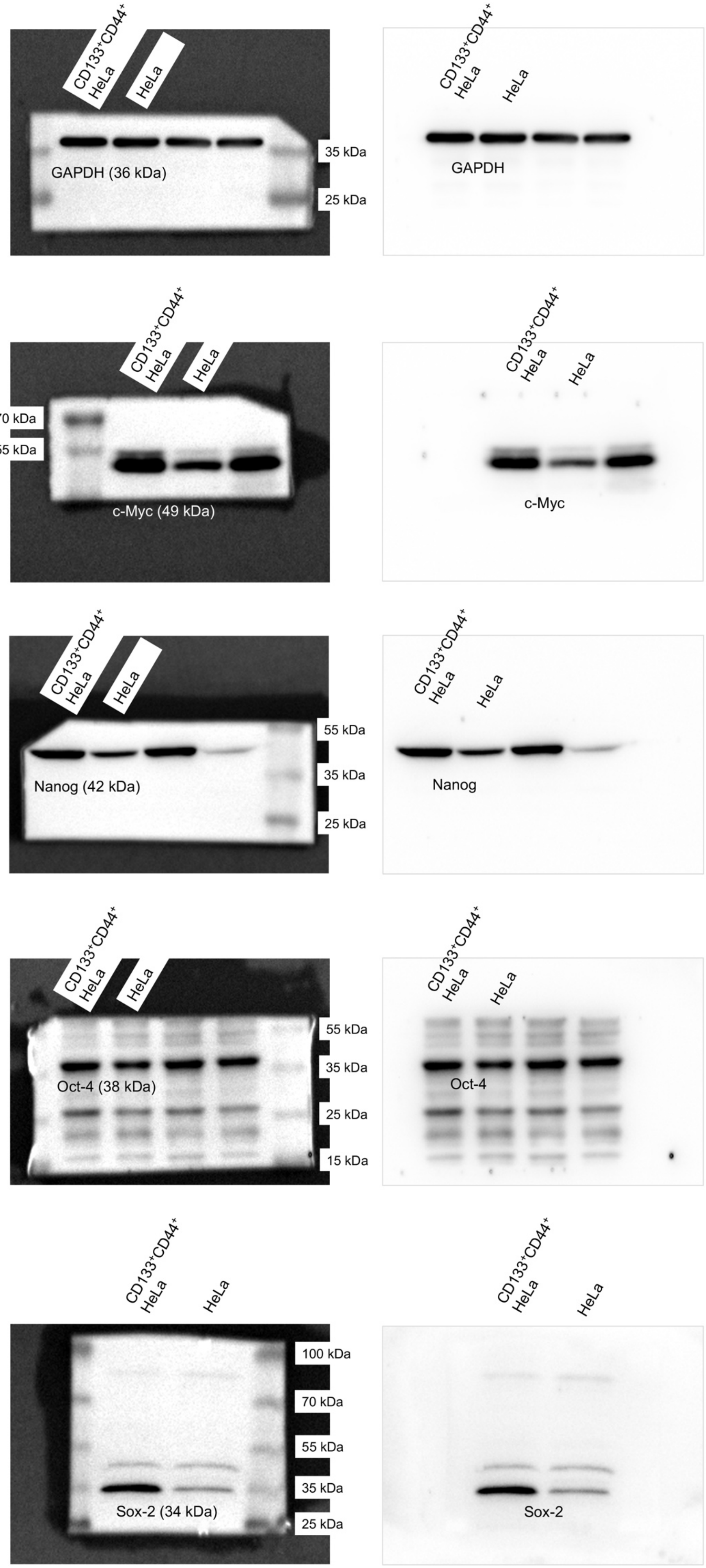
CD133+CD44+ HeLa
HeLa
35 kDa
25 kDa
GAPDH (36 kDa)
GAPDH
70 kDa
55 kDa
c-Myc (49 kDa)
c-Myc
55 kDa
35 kDa
25 kDa
Nanog (42 kDa)
Nanog
55 kDa
35 kDa
25 kDa
15 kDa
Oct-4 (38 kDa)
Oct-4
100 kDa
70 kDa
55 kDa
35 kDa
25 kDa
Sox-2 (34 kDa)
Sox-2

B

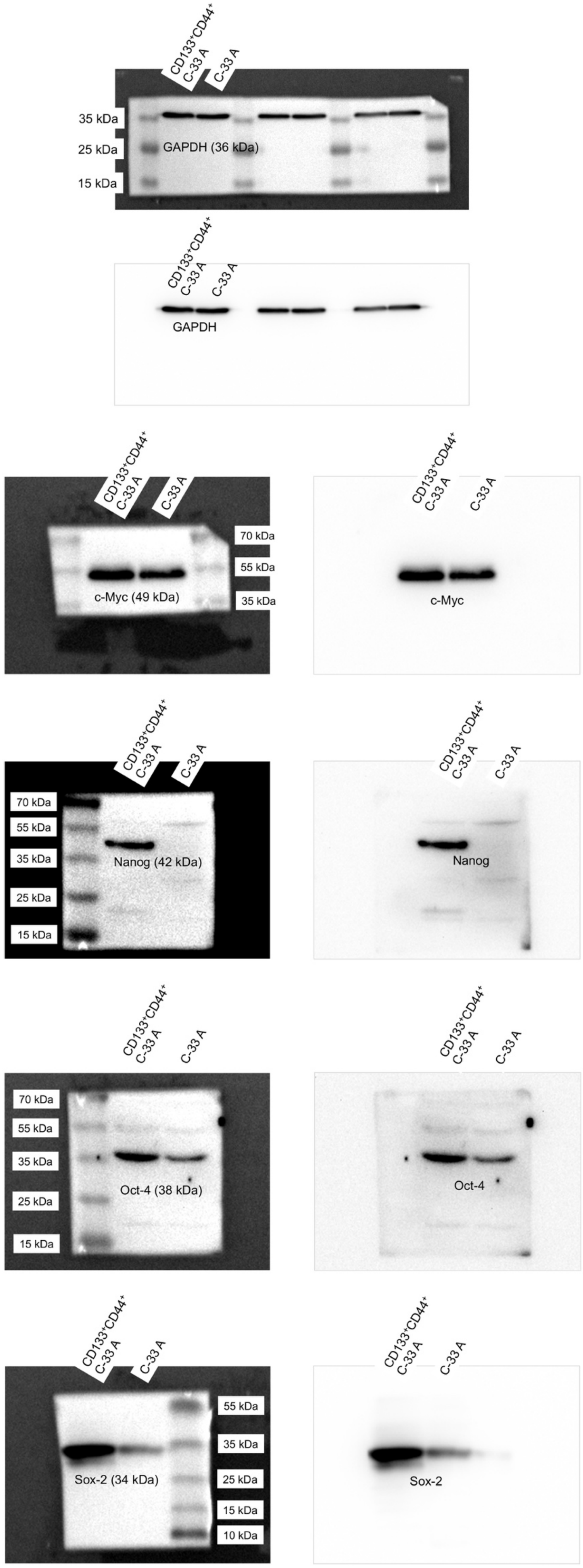
CD133+CD44+ C-33 A
C-33 A
35 kDa
25 kDa
15 kDa
GAPDH (36 kDa)
GAPDH
70 kDa
55 kDa
35 kDa
c-Myc (49 kDa)
c-Myc
70 kDa
55 kDa
35 kDa
25 kDa
15 kDa
Nanog (42 kDa)
Nanog
Oct-4 (38 kDa)
Oct-4
55 kDa
35 kDa
25 kDa
15 kDa
10 kDa
Sox-2 (34 kDa)
Sox-2

C

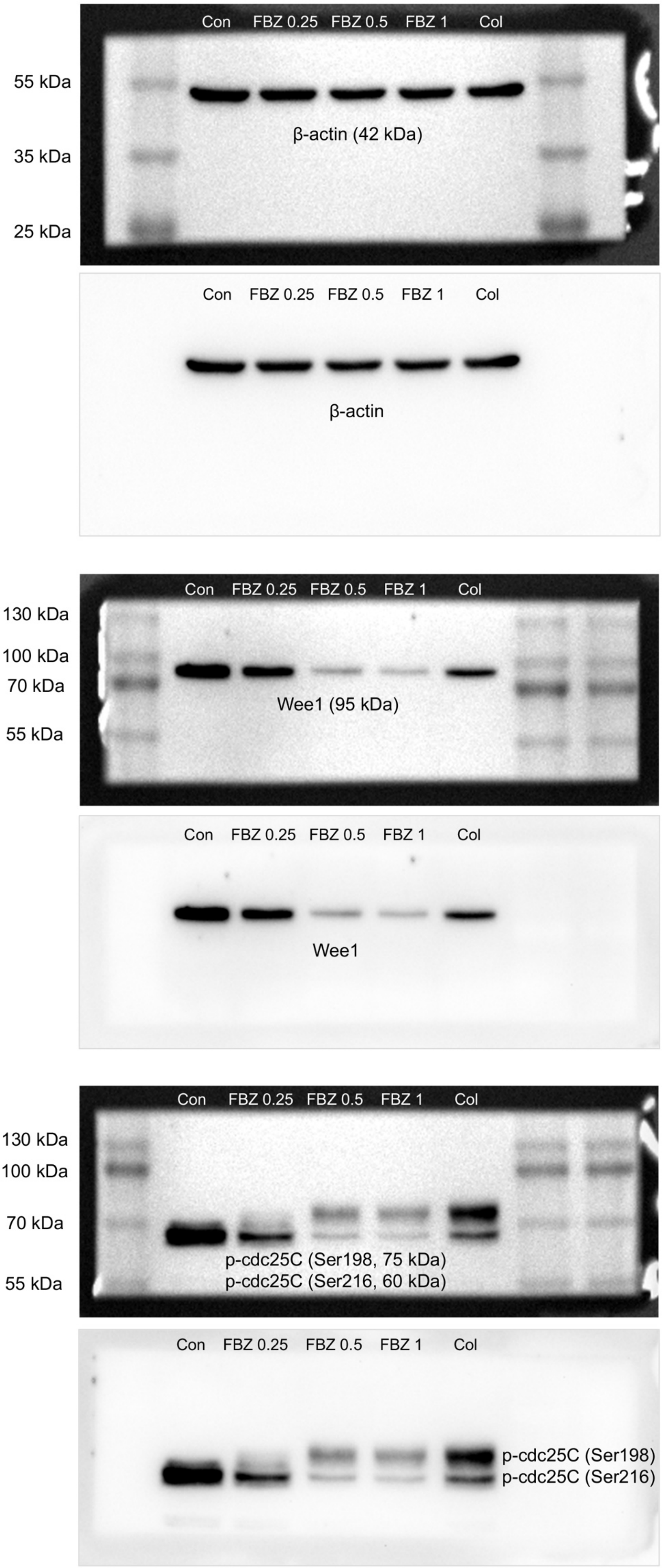
Con FBZ 0.25 FBZ 0.5 FBZ 1 Col
55 kDa
35 kDa
25 kDa
β-actin (42 kDa)
Con FBZ 0.25 FBZ 0.5 FBZ 1 Col
β-actin
Con FBZ 0.25 FBZ 0.5 FBZ 1 Col
130 kDa
100 kDa
70 kDa
55 kDa
Wee1 (95 kDa)
Con FBZ 0.25 FBZ 0.5 FBZ 1 Col
Wee1
Con FBZ 0.25 FBZ 0.5 FBZ 1 Col
130 kDa
100 kDa
70 kDa
55 kDa
p-cdc25C (Ser198, 75 kDa)
p-cdc25C (Ser216, 60 kDa)
Con FBZ 0.25 FBZ 0.5 FBZ 1 Col
p-cdc25C (Ser198)
p-cdc25C (Ser216)

D

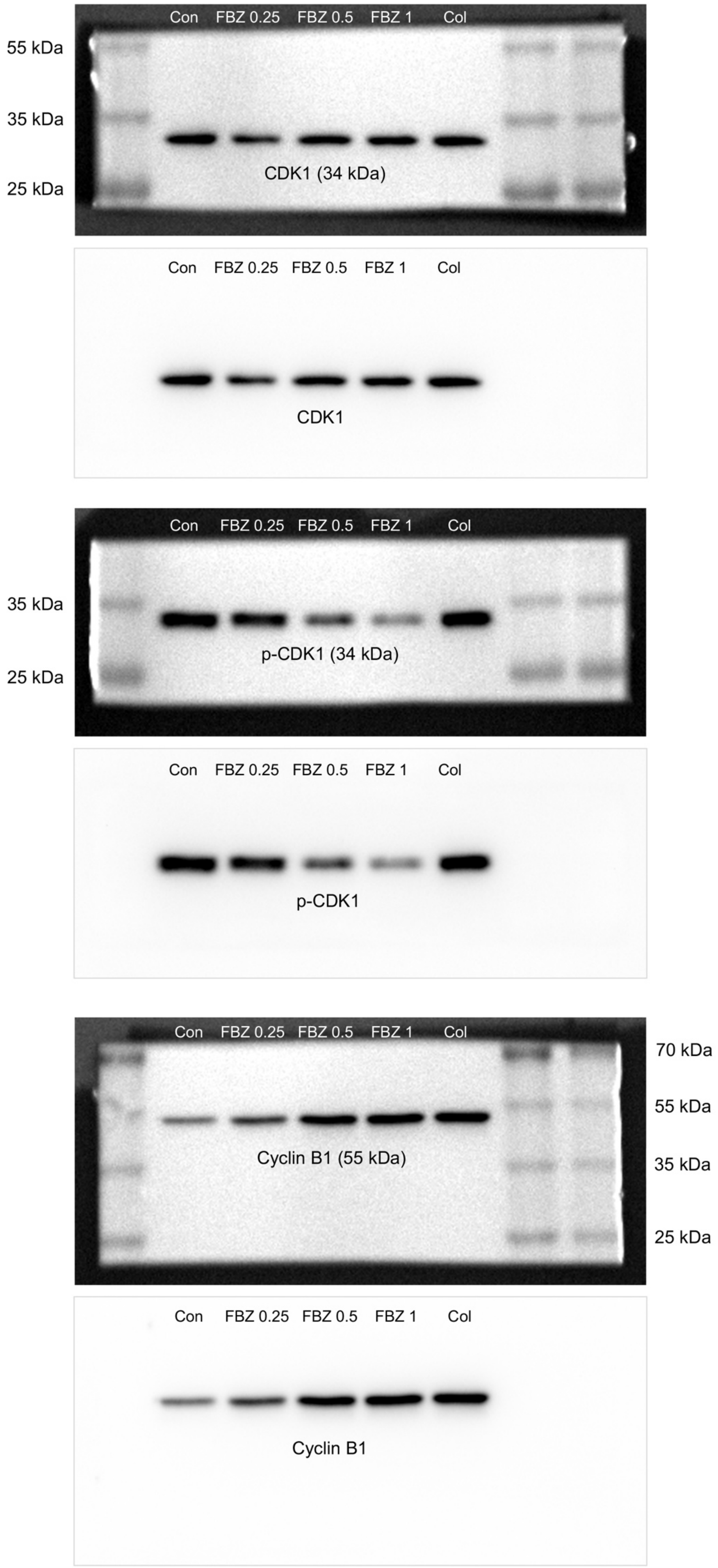

Con
FBZ 0.25
FBZ 0.5
FBZ 1
Col
55 kDa
35 kDa
25 kDa
CDK1 (34 kDa)
Con
FBZ 0.25
FBZ 0.5
FBZ 1
Col
CDK1
Con
FBZ 0.25
FBZ 0.5
FBZ 1
Col
35 kDa
25 kDa
p-CDK1 (34 kDa)
Con
FBZ 0.25
FBZ 0.5
FBZ 1
Col
p-CDK1
Con
FBZ 0.25
FBZ 0.5
FBZ 1
Col
70 kDa
55 kDa
35 kDa
25 kDa
Cyclin B1 (55 kDa)
Con
FBZ 0.25
FBZ 0.5
FBZ 1
Col
Cyclin B1

E

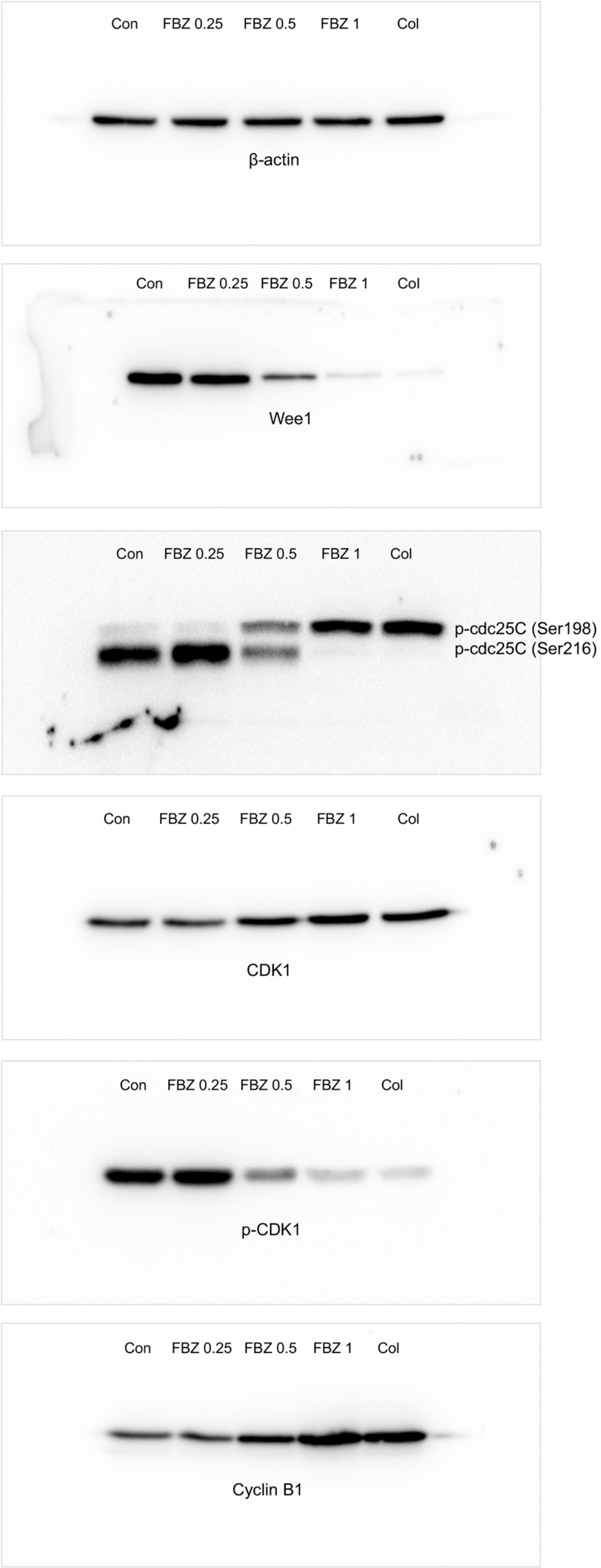
Con
FBZ 0.25
FBZ 0.5
FBZ 1
Col
β-actin
Con
FBZ 0.25
FBZ 0.5
FBZ 1
Col
Wee1
Con
FBZ 0.25
FBZ 0.5
FBZ 1
Col
p-cdc25C (Ser198)
p-cdc25C (Ser216)
Con
FBZ 0.25
FBZ 0.5
FBZ 1
Col
CDK1
Con
FBZ 0.25
FBZ 0.5
FBZ 1
Col
p-CDK1
Con
FBZ 0.25
FBZ 0.5
FBZ 1
Col
Cyclin B1

Figure S2. The original uncropped Western blot images corresponding to the results presented in Figures 1 and 3. (A) Detection of the stemness-related markers c-Myc, Nanog, Oct-4 and Sox-2 in $CD133^{+}CD44^{+}$ HeLa cells; (B) Detection of the stemness-related markers c-Myc, Nanog, Oct-4 and Sox-2 in $CD133^{+}CD44^{+}$ C-33 A cells; (C) Detection of the cell cycle associated proteins Wee1 and p-cdc25C in HeLa SCs; (D) Detection of the cell cycle associated proteins CDK1, p-CDK1 and cyclin B1 in HeLa SCs; (E) Detection of the cell cycle associated proteins Wee1, p-cdc25C, CDK1, p-CDK1 and cyclin B1 in HeLa cells.
