# Supplementary material for: Fenbendazole Exhibits Antitumor Activity Against Cervical Cancer Through Dual Targeting of Cancer Cells and Cancer Stem Cells: Evidence from In Vitro and In Vivo Models
Source: Molecules. 2025 May 29;30(11):2377. doi: 10.3390/molecules30112377 (PMC12156427; doi:10.3390/molecules30112377)
Supplement: Supplementary file 1 [file molecules-30-02377-s001.zip › Figure S3.pdf]

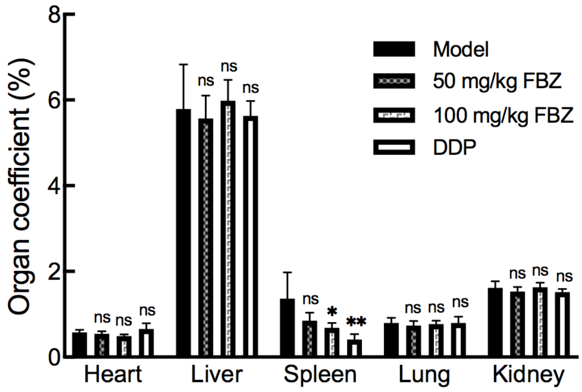

Figure S3. The organ coefficients of mice in each group after treatment. At the end of the treatment period, all mice were euthanized, followed by body weight measurement and collection of major organs (hearts, livers, spleen, lungs, and kidneys) for wet weight determination and subsequent calculation of organ coefficients (organ-to-body weight ratios). ∗: $p<0.05$ vs Model; ∗∗: $p<0.01$ vs Model; ns: no significance vs Model; n=5.
